# Absolute abundance calculation enhances the significance of microbiome data in antibiotic treatment studies

**DOI:** 10.3389/fmicb.2025.1481197

**Published:** 2025-03-24

**Authors:** Stefanie Wagner, Michael Weber, Lena-Sophie Paul, Angelika Grümpel-Schlüter, Jeannette Kluess, Klaus Neuhaus, Thilo M. Fuchs

**Affiliations:** ^1^Institute of Molecular Pathogenesis, Friedrich-Loeffler-Institut, Jena, Germany; ^2^Institute of Animal Nutrition, Friedrich-Loeffler-Institut, Braunschweig, Germany; ^3^Core Facility Microbiome, ZIEL Institute for Food & Health, Technical University of Munich, Freising, Germany

**Keywords:** antibiotic, one health, gut microbiome, piglet, flow cytometry, spike-in, 16S rRNA gene sequencing

## Abstract

**Background:**

The intestinal microbiota contributes to the colonization resistance of the gut towards bacterial pathogens. Antibiotic treatment often negatively affects the microbiome composition, rendering the host more susceptible for infections. However, a correct interpretation of such a perturbation requires quantitative microbiome profiling to reflect accurately the direction and magnitude of compositional changes within a microbiota. Standard 16S rRNA gene amplicon sequencing of microbiota samples offers compositional data in relative, but not absolute abundancies, and the presence of multiple copies of 16S rRNA genes in bacterial genomes introduces bias into compositional data. We explored whether improved sequencing data analysis influences the significance of the effect exerted by antibiotics on the faecal microbiota of young pigs using two veterinary antibiotics. Calculation of absolute abundances, either by flow cytometry-based bacterial cell counts or by spike-in of synthetic 16S rRNA genes, was employed and 16S rRNA gene copy numbers (GCN) were corrected.

**Results:**

Cell number determination exhibited large interindividual variability in two pig studies, using either tylosin or tulathromycin. Following tylosin application, flow cytometry-based cell counting revealed decreased absolute abundances of five families and ten genera. These results were not detectable by standard 16S analysis based on relative abundances. Here, GCN correction additionally uncovered significant decreases of *Lactobacillus* and *Faecalibacterium*. In another experimental setting with tulathromycin treatment, bacterial abundance quantification by flow cytometry and by a spike-in method yielded similar results only on the phylum level. Even though the spike-in method identified the decrease of four genera, analysis by fluorescence-activated cell sorting (FACS) uncovered eight significantly reduced genera, such as *Prevotella* and *Paraprevotella* upon antibiotic treatment. In contrast, analysis of relative abundances only showed a decrease of *Faecalibacterium* and *Rikenellaceae* RC9 gut group and, thus, a much less detailed antibiotic effect.

**Conclusion:**

Flow cytometry is a laborious method, but identified a higher number of significant microbiome changes in comparison to common compositional data analysis and even revealed to be superior to a spike-in method. Calculation of absolute abundances and GCN correction are valuable methods that should be standards in microbiome analyses in veterinary as well as human medicine.

## Introduction

Antibiotic treatment often has a detrimental impact on the gut microbiome integrity, resulting in an increased risk for infection (Stecher et al., 2013; Prax et al., 2021), and an improved functional understanding of this dysbiosis requires a proper analysis of the intestinal microbiota composition. However, 16S sequencing data is generally not fully representative of community composition, due to sampling, DNA isolation, primer choice, 16S rRNA gene copies, and data analysis (Abellan-Schneyder et al., 2021). Concerning data analysis, filtering of spurious taxa and primer trimming seems to have a major impact (Reitmeier et al., 2021; Haider et al., 2024). Another major limitation is that high-throughput 16S rRNA gene sequencing of microbiota samples provides compositional data that appear as relative instead of absolute abundancies. Relative abundancies quantify the different microbial taxa as fractions within a sample irrespective of its total cell numbers. Such relative microbiome profiling (RMP) often results in artefacts with respect to comparative taxon counts. In particular, a comparative analysis does not yield data about extent or directionality of compositional changes of a microbiota upon perturbation. For example, antibiotic treatment that decreases cells belonging to a specific microbial family necessarily results in an apparent increase of the relative abundance of a resistant family when RMP is applied. This hampers the identification of microbial taxa that are significantly affected upon intervention (Jian et al., 2020). Further drawbacks of describing relative abundances were stated in numerous publications (Vandeputte et al., 2021; Vandeputte et al., 2017; Galazzo et al., 2020; Lambrecht et al., 2017; Rao et al., 2021), but they have rarely been assessed in next generation sequencing (NGS) studies on microbiomes (Boshuizen and Te Beest, 2023).

To address this issue, microbial cell numbers of a sample need to be quantified by internal standards. For example, known amounts of DNA can be spiked into microbial samples before DNA extraction (Tkacz et al., 2018; Lin et al., 2019), an approach termed internal standard normalization (ISN) that has been established for quantitative and metagenome analysis (Satinsky et al., 2013). Using a set of environmental samples, Lin *et al*. demonstrated that community profiles and taxon co-occurrence patterns obtained by ISN substantially differed from RMP (Lin and Peddada, 2020). Another option is to spike a sample with a known number of exogeneous bacteria to adjust the microbiome composition (Stämmler et al., 2016). As an alternative, quantitative microbiome profiling (QMP) by qPCR, which targets 16S rRNA genes, is cost-effective, feasible and directly comparable to NGS (Jian et al., 2020). Challenges encountered here are the choice of a reference organism required to construct a standard curve, DNA extraction efficiencies, and the variance of strain-specific16S rRNA operon copy numbers per genome (Bonk et al., 2018). Flow cytometry of cells stained with a fluorescent dye is another feasible method to enumerate bacterial cells. Vandeputte and colleagues (Vandeputte et al., 2017) established a workflow for QMP of 40 faecal samples of a study cohort by flow cytometry and thus demonstrated that the association between Crohn’s disease and a low-cell-count Bacteroides enterotype is an artefact due to RMP. However, when DNA-binding stains are used, the fluorescence intensity is directly related to the nucleic acid content of the sample, possibly resulting in a bias due to distinct genome lengths, physiological states of a cell, or a lack of reproducibility in staining and storage conditions that cause DNA to deteriorate (Prest et al., 2013; Kamiya et al., 2007). Following a comparison of qPCR and flow cytometry, Galazzo et al. (2020) concluded that qPCR-based QMP is too imprecise to be an alternative to flow cytometry. In contrast, Jian and colleagues pointed out that microbiota sequenced by 16S rRNA amplicon sequencing differs from microbiota quantified by flow cytometry, because the DNA extracted from a faecal sample does not necessarily correlate with intact bacterial cells (Jian et al., 2020).

A further bias in microbiome analysis is introduced by up to 15 copies of 16S rRNA genes in a single genome (Angly et al., 2014; Vetrovsky and Baldrian, 2013). Bacteria with more than one copy of the 16S rRNA gene appear overrepresented as multiple sequences are attributed to single cells. Variations in 16S rRNA gene copy numbers (GCN) are particularly common in the phylum Bacillota and the class Gammaproteobacteria, which belongs to the phylum Pseudomonadota (Vetrovsky and Baldrian, 2013; Göker and Oren, 2024; Williams and Kelly, 2013). Although the exact number of the 16S rRNA gene is usually taxon-specific, variations among strains of the same species were also observed (Acinas et al., 2004).

In this study, we examined whether an optimized microbiota analysis of faeces samples from animals treated with antibiotics reveals significant effects that were not detected by RMP. The veterinary antibiotics tylosin and tulathromycin were administered to piglets in two independent animal trials. A correction of relative frequencies of bacterial taxa determined via NGS was performed by considering the 16S rRNA GCN. Absolute taxon abundancies were calculated for each taxon by measuring total bacterial cell numbers via by flow cytometry. For method comparison, cell numbers of samples from animals treated with tulathromycin were additionally determined using a spike-in method according to Tourlousse et al. (2017, 2018).

## Methods

### Piglet feeding trial A with tylosin application

Four weeks old female pigs obtained from the Mörsdorfer Agrar GmbH (Mörsdorf, Thuringia, Germany) were maintained in the animal facility of the Friedrich-Loeffler-Institute (Jena, Germany) in separate pens. After 2 weeks of acclimatization, piglets (*n* = 2 per group in pre-trial, *n* = 4 per group in main trial, 12 animals in total, 10.77 ± 1.39 kg live weight) were fed about 5 g of either pure peanut butter (Netto American Style, Netto Marken-Discount Stiftung & Co. KG, Maxhütte-Haidhof, Germany) or peanut butter supplemented with tylosin tartrate (Sigma-Aldrich Chemie GmbH, Taufkirchen, Germany) at a concentration of 10 mg/kg bodyweight per piglet. Each feeding was done twice in an interval of 24 h. Individual faecal samples were collected before (d0) and 30 h (d1), 48 h (d2), 72 h (d3), and 96 h (d4) after antibiotic treatment. Samples were homogenized, and aliquots were stored either at room temperature (RT) in 600 μL DNA stabilization solution (INVITEK Molecular, Berlin, Germany) for sequencing, or at −20°C without additives.

### Piglet feeding trial B with tulathromycin application

Eighty weaned barrows (6.39 ± 1.1 kg live weight) were group-housed (four piglets/pen) and equally assigned to one of four diets with graded copper levels (five pens/diet) during 5 weeks of rearing. At the end of the fourth experimental week, piglets of each dietary group were subdivided into half and were subjected to intramuscular injection of the antibiotic (DRAXXIN^®^ Zoetis, 2.5 mg tulathromycin/kg BW) or a placebo (0.9% NaCl). Individual faecal samples obtained via manual rectal stimulation were collected directly before and 24 h after the respective injection. Faecal material was snap-frozen in liquid nitrogen immediately after collection and stored at −80°C until further processing. Six piglets (antibiotic-treated individuals) of the dietary groups with 150 mg Cu/kg feed were chosen for further analysis in this study for total bacterial counts and sequencing.

### Sequencing and raw read processing

Isolation of total DNA and sequencing of 16S rRNA gene amplicons was carried out at the Core Facility Microbiome of the Technical University of Munich (Freising, Germany) as described previously (Reitmeier et al., 2020) with slight modifications. Briefly, DNA was isolated using a MaxWell (Promega, Walldorf, Germany) after bead-beating and used in a 2-step PCR to generate sequencing libraries. The first PCR used primers specific for the V3 and V4 regions (i.e., 341F, CCT ACG GGN GGC WGC AG; 785R, GAC TAC HVG GGT ATC TAA TCC) that contain an overhang for the subsequent PCR for sample barcoding. Cleaned libraries were sequenced PE300 on a MiSeq (Illumina). Spike-in of synthetic full-length 16S rRNA genes was done as described by Tourlousse et al. (2018, 2017). Here, 6 ng of spike DNA, consisting of an equimolar mixture of 13 linearized plasmids, each of which contains an artificial 16S rRNA gene, was added to 600 μL of the faeces-stabilizer mix. In each artificial “gene”, the invariant regions of the 16S rRNA were left untouched, while the variable regions were swapped with artificial sequences. Thus, spike reads are clearly distinguishable from true bacterial reads in analysis. Sample weight (i.e., gram of faecal material) was recorded in order to obtain 16S rRNA GCN per gram sample.

Raw reads were processed with pipeline DADA2 (Callahan et al., 2016). Sequences were demultiplexed and filtered, and amplicons with an expected error > 2 were excluded. To limit the analysis of regions with higher error values, reads were trimmed to sequence lengths of 250 bp and 200 bp, respectively, for forward and reverse reads. Remaining reads were merged to paired end reads. Amplicon sequence variants (ASVs) were clustered at 97% sequence identity, and their sample-wise abundances were calculated after removing substitution and chimera errors. Taxonomies were assigned at 80% confidence level by considering results from both the Ribosomal Database Project (RDP) classifier (Wang et al., 2007) and the SILVA Incremental Aligner (SINA; v1.2.11) (Pruesse et al., 2012). Taxon names were verified manually in accordance to the nomenclature defined by the List of Prokaryotic names with Standing in Nomenclature (LPSN) (Parte, 2013; Parte, 2018; Parte et al., 2020; Euzéby, 1997).

### 16S rRNA GCN correction and synthetic spike-in

ASVs were analysed using parts of the PICRUSt2 pipeline (Douglas et al., 2020) as follows. HMMER version v3.3.2^1^ places ASVs, EPA-ng (Barbera et al., 2018) determined the optimal position of these ASVs in a reference phylogeny, and GAPPA (Czech and Stamatakis, 2019) outputs a new tree incorporating ASV placements. This adjusted reference phylogeny allowed for the prediction of 16S rRNA GCN. The IMNGS output ASVs tables were corrected within the PICRUSt2 pipeline by dividing the original read counts by the predicted GCN.

To obtain a more intuitive comparability of both methods, we harmonized absolute abundances obtained by a flow cytometry method, FACS, and the spike-in approach as follows. Since the spike-in method gives only relative numbers of 16S rRNA gene copies between samples, cell counts of piglet 7 determined by FACS on day 0 were used as a reference. Subsequently, read counts for the spikes of this sample were scaled to the cell numbers within this sample using an arbitrary factor. The factor was chosen such that the relative amount of spike in this sample could be converted into the cell number found by FACS. The other samples gave relative numbers of gene copies that were multiplied by this factor in order to calculate cell number equivalents.

### Flow cytometry measurements

Frozen faecal samples were split into 0.1-g aliquots in triplicate and slowly thawed on ice. Aliquots were diluted in 10 mL 0.85% (w/v) NaCl and homogenized for 3 min with a Vortex-Genie 2 mixer (Scientific Industries, New York, United States). To remove faecal debris, the solutions were filtered using a sterile syringe filter with 5 μm pore size (Macherey-Nagel, Düren, Germany). Next, 500 μL of the filtered cell suspension were mixed with three volumes of fixation buffer (4% paraformaldehyde, 200 nM Na_2_HPO, pH 7.2) for at least 3 hours at RT. Subsequently, the samples were centrifuged at 12,000 × g for 10 min, and the supernatant was discarded. The remaining pellets were dissolved in 500 μL sterile filtered PBS (Sigma-Aldrich, Steinheim, Germany) and stained using the LIVE/DEAD™ *Bac*Light™ kit (Invitrogen, Karlsruhe, Germany).

Quantification of microbial cells in the faecal suspensions was performed using a FACS Canto II flow cytometer (BD Biosciences, NJ, United States). Fluorescence events were monitored using 530 nm and 660 nm optical detectors. Forward-and sideward-scattered light was also collected. The BD FACSDiva™ Software and FlowJo (both BD Biosciences) were used to gate and separate the microbial fluorescence events on the FITC-PE density plot from the faecal sample background. The gated fluorescence events were evaluated on the forward-sideways density plot to exclude remaining background events and to obtain an accurate microbial cell count. Instrument and gating settings were identical for all samples. Measurements were conducted in triplicates.

### Integration of cell counts into relative abundances

To calculate absolute frequencies of individual taxa, flow cytometry-measured bacterial cell counts were integrated into the ASV table created with DADA2 (Callahan et al., 2016). For this purpose, the read counts of each taxon in a sample were divided by the total read count of that sample. Subsequently, these numbers were multiplied by the bacterial cell count of the sample. The sum of all taxa in a sample yielded the total bacterial cell counts.

### Statistical analysis

All further analyses were performed in the R programming environment using Rhea (Lagkouvardos et al., 2017), following scripts and instructions available online.^2^ A PERMANOVA test (vegan::adonis) was performed in each case to determine if the separation of sample groups was significant, as a whole and in pairs. For the analysis of relative abundances, counts were standard normalized using total sum scaling. To analyse absolute abundances, no normalization was applied after integration of bacterial cell counts. The filtered and, in case of relative abundances, normalized ASVs table used as basis for all analyses is provided in Supplementary Tables 1, 2. *α*-diversity was computed based on generalized UniFrac distances (Chen et al., 2012). *β*-diversity was assessed on the basis of species richness and Shannon effective diversity (Jost, 2007) as explained in detail in Rhea. *p* values were corrected for multiple comparisons according to the Benjamini-Hochberg method. Only taxa with a prevalence ≥30% (proportion of samples positive for the given taxa) in one given group and relative abundance ≥0.25% (Reitmeier et al., 2021) in at least one sample were considered for statistical testing. Statistical analyses were performed as described for each experiment and *p* values ≤0.05 were considered as significant.

## Results and discussion

### Relative and absolute bacterial abundances upon tylosin treatment of piglets

**Figure 1:**
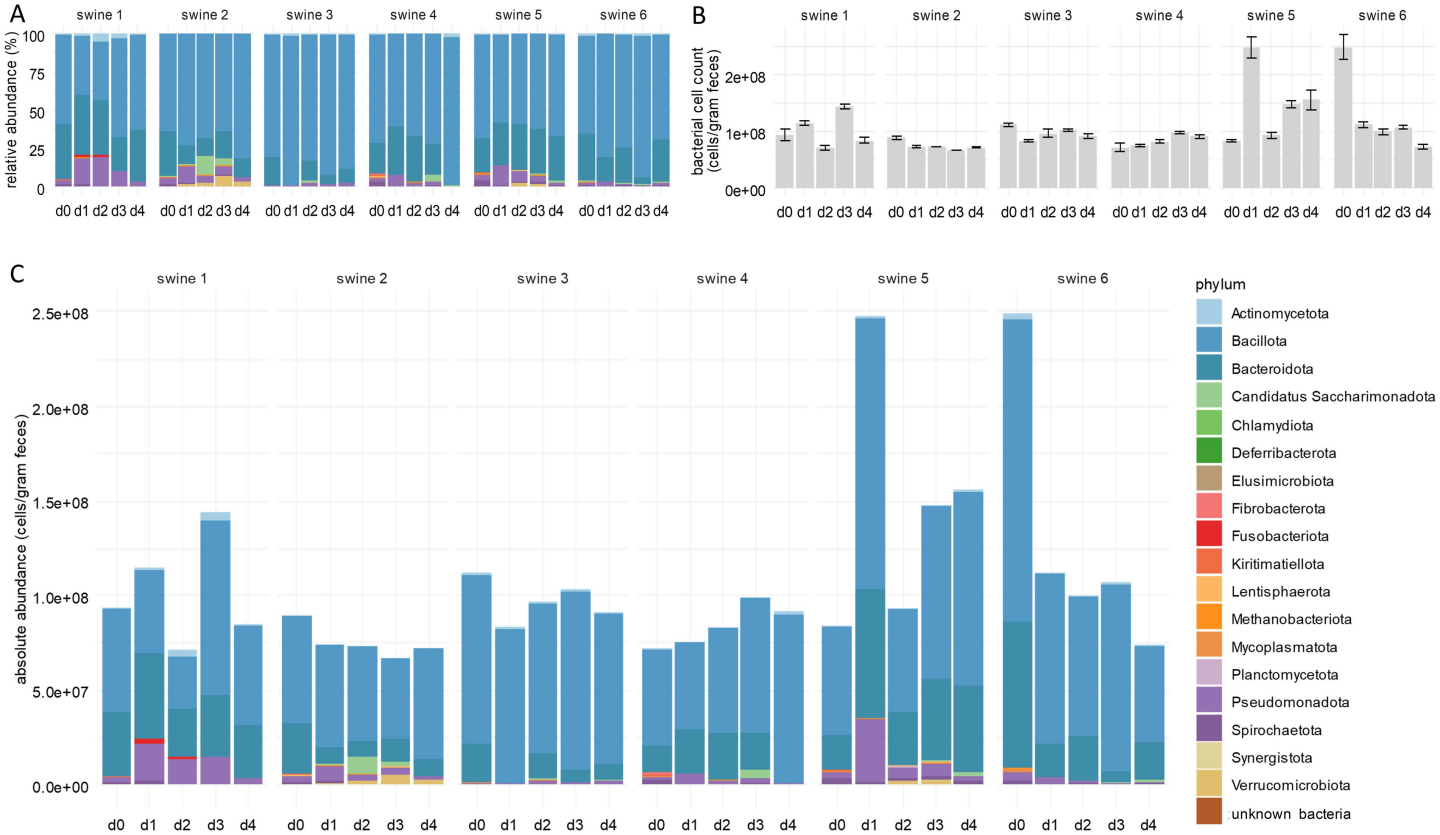
Integration of bacterial cells counts into faecal microbiome composition. **(A)** Relative abundances of bacterial phyla deduced from standard microbiome 16S rRNA gene amplicon sequencing. **(B)** Bacterial cell counts per gram faeces measured by flow cytometry. **(C)** Absolute abundances of bacterial phyla following integration of bacterial cell counts into 16S rRNA gene sequencing data. Per phylum, cumulative abundances were calculated from all single ASV classified within one phylum using both RDP and SILVA. The numbers above the bars indicate individual piglets. Samples d0–d4 were taken immediately before and at the 4 days following tylosin treatment. Bar plots for individual piglets are shown (*n* = 6).

In the first experimental setting, 10 mg of tylosin per kg bodyweight was applied twice in a 24 h-interval orally to six animals. Faecal samples were collected immediately before (sample d0) and at day 1 to 4 (samples d1–d4) after application. To investigate effects of tylosin on the composition of the piglet microbiota, we performed 16S rRNA gene amplicon sequencing of faecal samples. Statistical analysis revealed no significant changes in the faecal microbiota compositions of control group piglets, whereas tylosin treatment caused various effects. In more detail, the *α*-diversity of faecal microbiota compositions on day 1 to 4 of each tylosin treated animal in comparison with day 0 was calculated for each animal (Supplementary Figure 1). Throughout the whole group, the number of species and the Shannon effective number significantly decreased after tylosin application, as previously described for other macrolide antibiotics such as azithromycin (McDonnell et al., 2021). Despite an overall reduction in species richness following tylosin treatment, a significant increase in the relative abundance of Pseudomonadota was observed post-application (Figure 1A; Supplementary Table 3; Supplementary Figure 2). The abundance of this phylum increased in the microbiota of all animals tested here, but only in one piglet to a considerable extent. In sample d3, we observed a marked increase in the abundance of Bacillota, indicating a microbiota rebalancing post-tylosin disturbance, while the abundance of Pseudomonadota decreased. Four days after application, the microbiota composition closely resembled that of sample d0, with a minor increase in the abundance of Bacillota.

The values gained by RMP, however, do not necessarily mirror absolute cell numbers of a taxon present in a sample. For example, the true reduction of a taxon can result in an apparent decrease of the total cell count without affecting absolute frequencies of the other taxa. To overcome this limitation of RMP, we applied flow cytometry to all samples analysed above. Total cell numbers determined at day 0 ranged from 7.20 × 10^7^ cells per gram faeces (c/gf) to 2.49 × 10^8^ c/gf, with the group median of 9.15 × 10^7^ c/gf (Figure 1B; Supplementary Table 4). Along the experimental course, the median number remained nearly constant. Total cell counts not only varied among the animals but also within an individual over time due to cell densities in faeces depending on water content and other physiological factors (Vandeputte et al., 2021; Wang et al., 2007). Nevertheless, results from individual samples of the present study are comparable to each other upon careful sample processing, even if they deviate from the results of other studies (Vandeputte et al., 2021; Vandeputte et al., 2017; Wang et al., 2007). The method used in this study differs from other protocols by an additional fixation and washing step, possibly resulting in a reduced number of bacteria.

### Integration of bacterial cell counts into faecal microbiome composition analysis improves the explanatory power of a porcine faecal microbiome analysis

Total cell counts obtained by flow cytometry were integrated into the relative abundances of bacterial taxa as determined by 16S rRNA gene amplicon sequencing. The resulting absolute abundances revealed that the increase of Pseudomonadota, detected by RMP, was not an artefact due to limitations of this method (Figure 1C; Supplementary Table 3; Supplementary Figure 2), but indicated an actual bloom of this phylum. This observation can be explained by the inhibition of other phyla by tylosin, thus creating ecological niches that favour the spread of Pseudomonadota (Morton et al., 2019), in turn contributing to dysbiosis, intestinal diseases, and increased susceptibility towards infections (Shin et al., 2015; Sun et al., 2019; Bonardi, 2017; Bin et al., 2018).

### Correction of 16S rRNA GCN increases the accurateness of family-and genus-level analysis

To reduce bias due to 16S rRNA gene copies present in a bacterial genome, we determined the GCN for each molecular species using PICRUSt2 (Douglas et al., 2020) and corrected relative frequencies accordingly. This pipeline has the advantage that it predicts GCN of unknown genera based on similarities to already known sequences.

**Figure 2:**
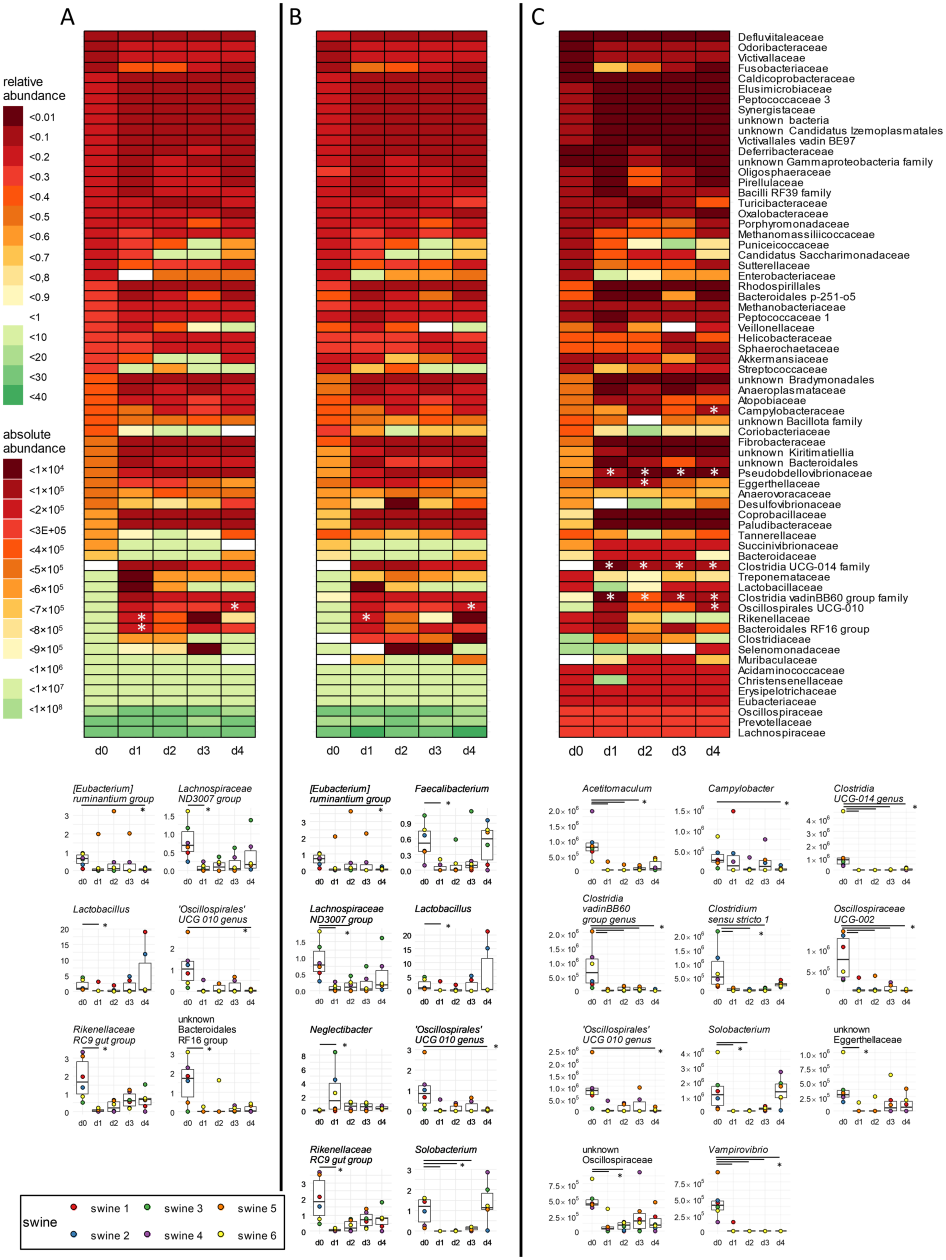
Correction of 16S rRNA GCN and integration of bacterial cell counts in family-and genus-level analyses. **(A)** Relative abundances were obtained by standard analysis of 16S rRNA gene sequencing data. **(B)** Same data set as **(A)**, but corrected for GCN of the 16S rRNA genes. **(C)** Same data as in **(B)**, but absolute abundances were obtained by integration of bacterial cells counts via flow cytometry. Heat maps for the occurrence of families over time points are shown as mean values over all six animals. Below each heat map, significantly changed relative and absolute abundances of single genera are shown in boxplots. Cumulative abundances were calculated from all single ASVs classified within one family or genus as per the best possible taxonomy using both RDP and SILVA. * *p* < 0.05 pairwise Fisher’s Exact Test, *n* = 6.

Of a total of 234 genera, 63 genera (26.9%) were identified in our samples to harbour more than one copy of the 16S rRNA gene in the genome, and most of them carried up to five copies (Supplementary Table 5). Genus *Pseudescherichia* exhibited six copies, genus *Clostridium* sensu stricto 6 eight copies, and genus *Paenibacillus* even nine copies, indicating that the significance of these individual taxa for the overall microbiota composition is overestimated. Following GCN correction of relative abundances, the decrease observed for the families *Rikenellaceae* and Oscillospirales UCG-10 was confirmed (Figures 2A,B top; Supplementary Table 6). On genera level, GCN correction decreased the proportion of taxa with more than one copy of the 16S rRNA gene and thus influenced results of the statistical analysis (Figures 2A,B, bottom). The decrease in unknown *Bacteroidales* RF16 group was not confirmed by GCN correction, which is consistent with the observations already made at the family level. The relative abundance of some genera (i.e., *Lachnospiraceae* ND3007 group, *Lactobacillus*, the *Rikenellaceae* RC9 gut group, and an unknown Bacteroidales RF16 group) showed a decrease on day 1 compared to day 0 only after GCN correction. The *Lachnospiraceae ND3007* group is known to be positively correlated with a health supporting diet, which is rich in fibre and plant-based foods (Ericson et al., 2020; Ma et al., 2021). A restriction of fibre-degrading bacteria by antibiotics may have a negative effect on the energy production and thus on the growth performance of animals. *Lactobacillus* has been identified as one of the core genera in the gastrointestinal tract of pigs (Valeriano et al., 2017), contributing to overall health and growth performance and increasing the productivity of swine husbandry (Kenny et al., 2011; Yang et al., 2015). The *Rikenellaceae* RC9 gut group typically experiences an increase after weaning, coinciding with the transition of swine to solid food digestion (Saladrigas-García et al., 2022). In addition, significant decreases in the abundances of *Faecalibacterium*, *Neglectibacter*, and *Solobacterium* were overlooked due to missing GCN correction (Figures 2A,B, bottom). *Faecalibacterium* is a short chain fatty acids (SCFAs) producing genus with potential benefits for human health (Martin et al., 2023), thus underlining the relevance of GCN correction in microbiome analysis.

### Combination of absolute cell numbers and GCN reveals further hidden significant changes of bacterial abundances in tylosin-treated piglets

Integration of total cell counts into GCN-corrected relative abundances revealed several significant changes in different taxa, which were insignificant before. The decrease noted for *Rikenellaceae* by RMP was not confirmed by QMP upon data integration (Figure 2C top; Supplementary Table 6), in contrast to the decrease of the Oscillosporales UCG-10 family on day 4. Significant reductions were noted in the Clostridia UCG-014 family, in the Clostridia vadin BB60 group, and in *Pseudobdellovibrionaceae* from day 1 to day 4 post-treatment. Additionally, a significant decrease was recorded for the abundance of *Eggerthellaceae* on day 2 and of *Campylobacteraceae* on day 4. To summarize, relative abundances corrected with GCN only revealed significant decrease of two different families, while absolute abundance analysis of such data showed a significant change of six different bacterial families over time.

On the genus level, eleven different genera were found to have a significant decrease when absolute abundances together with GCN were considered (Figure 2C, bottom). The decrease of *Solobacterium*, an opportunistic pathogen, and the genus Oscillospirales UCG-010, as already observed upon RMP, was confirmed. In contrast, six different genera showed statistically significant changes in relative abundances, but did not exhibit significant changes in absolute analysis (Figures 2B,C, bottom). Additionally, *Campylobacter* displayed a decreased abundance on day 4 and an unknown genus from *Oscillospiraceae* on day 1 and day 2. *Acetitomaculum* and *Clostridium* sensu stricto 1 showed decreased abundance on days 1 to 3, and Clostridia UCG-014, Clostridia vadin BB60 group, *Oscillospiraceae* UCG-002, and *Vampirovibrio* exhibited decreasing abundances consistently over 4 days. *Oscillospiraceae* UCG-002 support breakdown of aspartate and glycine, and the unknown Clostridia UCG-014 facilitates degradation of tryptophan in the intestine (Atzeni et al., 2022; Yang et al., 2021). Both genera are important for the normal intestinal function of animals. The inhibition of SCFA-producing genera such as Clostridiales vadin BB60, the genus Oscillospirales UCG-010, and *Acetitomaculum*, may reverse the positive effects described above (Sawicka-Smiarowska et al., 2021; Sebastià et al., 2024; Greening and Leedle, 1989). The opportunistic pathogen *Clostridium* sensu stricto 1 was shown to be associated with inflammatory bowel disease and a reduced concentration of SCFA in the intestine (Yang et al., 2019; Hu et al., 2021). Inhibition of these bacteria can have positive effects on the health of animals by preventing relapsing infection (Bublitz et al., 2023).

To summarize the tylosin treatment data, correcting for 16S rRNA gene copies and the integration of bacterial cell counts increased the explanatory power of the data regarding such a perturbation (Kim et al., 2016; Candon et al., 2015; De La Cochetiere et al., 2005; Dethlefsen and Relman, 2011). Until now, any such correction has hardly been applied in microbiome studies. GCN combined with QMP not only revealed tylosin activity against opportunistic pathogens, but also stronger effects of the antibiotic on beneficial commensal bacteria otherwise not detectable by RMP. This result may explain a stronger impairment of functions of the gut microbiota such as the maintenance of colonization resistance and, thus, an increased probability of subsequent infections as compared to previous data (Collington et al., 1972; Kim et al., 2012).

### FACS and spike-in counting for QMP are equivalent methods on the phylum level

To compare two methods for calculating abundances, namely flow cytometry-based cell counting and spike-in of synthetic full-length 16S rRNA genes, we analysed the microbiota of samples from tulathromycin-treated piglets of a second experimental setting (see Supplementary Table 7 for an overview of the workflow). For comparability of both methods, we applied an arbitrary factor based on the sample from piglet 7 on day 0 as the reference (see method for details) that revealed cell number equivalents. Since FACS analyses yielded about 6.0 × 10^7^ c/gf, cell number equivalents for swine 7 using spike-in DNA were set to the same level.

**Figure 3:**
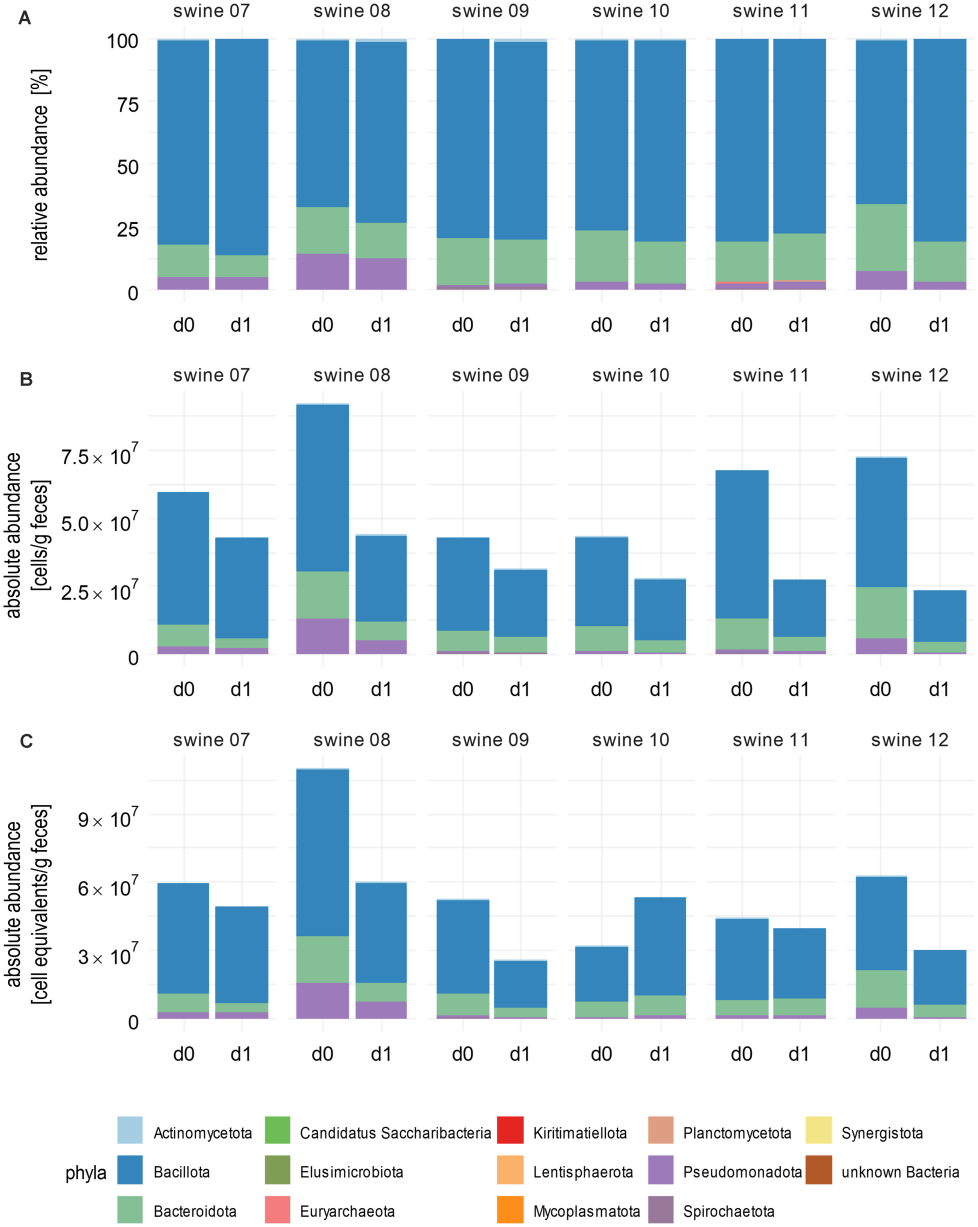
Absolute abundances of bacterial phyla after integrating total cells or spike-in counts. **(A)** Stacked bar plots show phyla abundances after correction for GCN of 16S rRNA genes. **(B)** Absolute abundancies as measured by flow cytometry and **(C)** as calculated after using spike-in DNA. Bacterial cell counts per gram faeces are shown. Cumulative abundances were calculated from all single ASVs classified within one phylum as per the best possible taxonomy using both RDP and SILVA. Numbers above the bars indicate individual piglets. Samples were taken before (d0) and 1 day after (d1) tulathromycin treatment (*n* = 6).

RMP of the samples from piglets treated with tulathromycin identified 15 different phyla (Figure 3A; Supplementary Table 8) and a decrease of Bacteroidota across most piglets that correlated with a relative increase of Bacillota. Piglets 9, 10, and 11 exhibited only minor changes at the phylum level, indicating a stable composition of their microbiomes. Incorporating total cell counts obtained by flow cytometry into RMP revealed a consistent decrease in total bacterial cell numbers across all piglets, ranging from 27% for piglet 9 to 68% for piglet 12 (Figure 3B; Supplementary Table 8). These differences, as well as the variation of initial total bacterial cell numbers, which range from 2.3 × 10^7^ to 9.2 × 10^7^ c/gf, underline the high variability of individual faecal microbiomes. In contrast to RMP, Bacillota exhibited a decrease from 4.8 × 10^7^ c/gf on day 0 to 1.9 × 10^7^ c/gf on day 1, which is a fold change [FC] of 0.40 after integration of bacterial cell counts. Similarly, a decrease in Bacteroidota was observed in all piglets, which contrasted with relative increases noted in the microbiome of piglet 11 due to RMP (FC = 1.15).

Next, we compared the results of FACS with those of the spike-in results. The changes of absolute abundancies on the phylum level as revealed by the spike-in method were mostly similar compared to results calculated with total cell counts (Figure 3C; Supplementary Table 8). An exception was observed in piglet 10, where an increase in total absolute abundances of Actinomycetota (1.5-fold), Bacillota (1.8-fold), and Spirochaetota (1.7-fold) was noted. Integration of spike-in sequencing data into GCN-corrected RMP revealed a decreased total cell count for most of the samples in line with flow cytometry-based analysis.

Taken together, the phyla reductions upon tulathromycin treatment observed in absolute abundances were more prominent than those observed for relative abundances.

### QMP by FACS is superior to spike-in on the family and genus level

**Figure 4:**
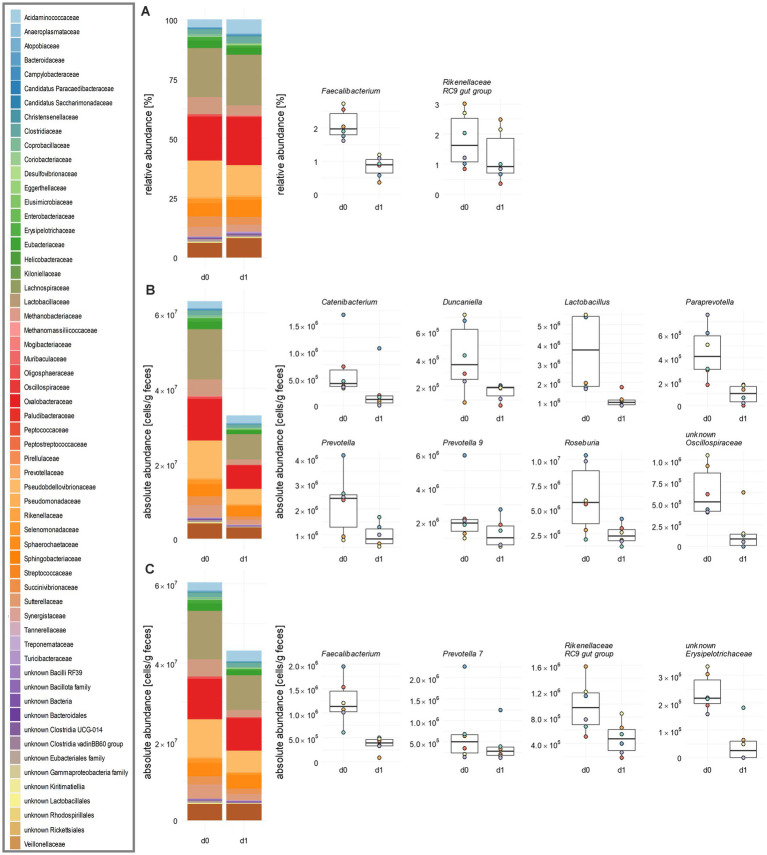
Influence of tulathromycin on the family and genus level of porcine faecal microbiota. **(A)** Relative abundances of bacterial families obtained by standard analysis of 16S rRNA gene sequencing data including GCN correction. **(B)** Same data as in **(A)** after integrating total cell counts obtained from flow cytometry and **(C)** after calculating absolute abundances of bacterial families from spike-in DNA after sequencing. In each panel, an overview is given by stacked bar plots for family abundances. Significantly changed relative and absolute abundances of single genera are shown in boxplots. Mean values over all six animals are indicated. Cumulative abundances were calculated from all single ASVs classified within one family as per the best possible taxonomy using both RDP and SILVA. All significant changes were indicated with the Paired Wilcoxon Signed Rank Sum Test with *p* ≤ 0.05 (*n* = 6).

The application of tulathromycin caused dynamic shifts of the abundance of families and genera. Among the 59 families detected by RMP, a relative decrease was observed in 33 families (Figure 4A; Supplementary Table 9; Supplementary Figure 3). For instance, *Rikenellaceae* experienced a significant relative decrease from 2.0 to 1.3%. This decline was associated with a relative increase in 26 other families, including *Tannerellaceae* and *Acidaminococcaceae.*

Integration of flow cytometry data revealed a decrease in mean cell counts from 6.32 × 10^7^ c/gf to 3.3 × 10^7^ c/gf after treatment (Figure 4B; Supplementary Table 9). In contrast to RMP, significant reductions in *Lachnospiraceae*, *Lactobacillaceae*, *Oscillospiraceae*, and *Prevotellaceae* across all piglets during the same time were observed. In contrast, when applying a data analysis using the spike-in method, we also found a decrease for 42 families, but none became significant (Figure 4C; Supplementary Table 9; Supplementary Figure 4). Sample analysis at the genus level revealed distinct trends in relative and absolute abundances. Upon RMP, *Faecalibacterium* decreased relatively from 2.10 to 0.84%, and the *Rikenellaceae* RC9 gut group from 1.81 to 1.25% (Figure 4A; Supplementary Figure 3). Integration of FACS data revealed eight genera significantly decreased with respect to absolute abundances, namely *Catenibacterium*, *Duncaniella*, *Lactobacillus*, *Paraprevotella*, *Prevotella*, *Prevotella 9*, *Roseburia*, and an unknown genus of *Oscillospiraceae* (Figure 4B; Supplementary Figure 3). For instance, there was a decrease in the key gut genera and SCFA-producer *Lactobacillus* and *Prevotella,* which contribute to the maintenance of the intestinal barrier, from 3.6 × 10^6^ to 1.1 × 10^6^ and from 2.2 × 10^6^ c/gf to 9.6 × 10^5^ c/gf, respectively (Supplementary Figure 4). While *Faecalibacterium* and *Rikenellaceae* RC9 gut group still decreased from day 0 to day 1, this decrease was no longer significant in pairwise tests.

Following analysis of spiked-in samples, *Faecalibacterium* and *Rikenellaceae* RC9 gut group were again found to be statistically significantly reduced, with *Faecalibacterium* decreasing by a FC of 0.29 and *Rikenellaceae* RC9 gut group by a FC of 0.05 (Figure 4C; Supplementary Figure 4). Additionally, the number of *Prevotella 7* was reduced by a FC of 0.6, and an unknown genus from *Erysipelotrichaceae* by a FC of 0.2. To summarize, the spike-in method yielded a higher number of significant changes of the microbiota on the family and genus level compared to RMP, but fewer effects than total cell counting by FACS.

## Conclusion

Integrating absolute cell counts into relative sequence data is one step further for accurately assessing gut microbiota states (Lin and Peddada, 2020), especially since changes in bacterial density may indicate various health conditions (Morjaria et al., 2019; Contijoch et al., 2019). Our study supports the significance of a precise determination of absolute cell numbers, either by flow cytometry analysis or spike-in of DNA, to avoid misinterpretation of microbiome data. The FACS approach, however, requires a greater effort in preparation than the spike-in method based only on adding appropriate amounts of synthetic DNA to the sample ahead of DNA isolation. We observed a high degree of comparability between the two methods to calculate absolute abundancies. Integration of total cell counts by FACS detected a larger number of significant changes in the compositional data of the microbiomes on the level of families and genera. Although there is potential for errors, for example due to high interindividual variance, the need of standards in cell quantitation, and the lack of comparability between samples to be sequenced on the one hand and the quantified microbiota on the other hand (Jian et al., 2020), the presented benefits of improved data analysis outweigh their drawbacks.

## Data Availability

The data presented in the study are provided by the Supplementary material and deposited in the SRA repository, accession numbers PRJNA1229264 and PRJNA800240, respectively.

## Footnotes

^1^ http://www.hmmer.org

^2^ https://lagkouvardos.github.io/Rhea/

## Supplementary material

SUPPLEMENTARY FIGURE 1 α-diversity of faecal microbiota upon tylosin treatment. **(A)** Normalized number of species and **(B)** Shannon effective numbers of faecal microbiota obtained by standard analysis of 16S rRNA gene sequencing data is shown a line plot. Individual piglets are indicated by the corresponding colours. Cumulative abundances were calculated from all single ASVs classified within one family as per the best possible taxonomy using both RDP and SILVA (# *p* < 0.05 after Paired Wilcoxon Signed Rank Sum Test, *n* = 6).

SUPPLEMENTARY FIGURE 2 Correction of 16S rRNA GCN and integration of bacterial cell counts into relative abundance analyses of the phylum Pseudomonadota. **(A)** Relative abundances of Pseudomonadota were obtained by standard analysis of 16S rRNA gene sequencing data. **(B)** Same data set as panel A, but corrected for GCN of the 16S rRNA genes. **(C)** Same data as in panel B, but absolute abundances of Pseudomonadota were obtained by integration of bacterial cells counts via flow cytometry. Relative and absolute abundances in the faecal microbiota of each animal are shown in boxplots. Cumulative abundances were calculated from all single ASVs classified within one phylum as per the best possible taxonomy using both RDP and SILVA (# *p* < 0.05 after Paired Wilcoxon Signed Rank Sum Test, *n* = 6).

SUPPLEMENTARY FIGURE 3 Influence of tulathromycin on the family level of porcine faecal microbiota after integrating total cells or spike-in counts. Relative abundances of bacterial families were obtained by standard analysis of 16S rRNA gene sequencing data including GCN correction (A columns). The same data set as in A columns is shown after integrating total cell counts obtained from flow cytometry (B columns) and after calculating absolute abundances of bacterial genera from spike-in DNA after sequencing (C columns). Heat maps for the abundance fold change of families following tulathromycin treatment are shown as mean values over all six animals. Cumulative abundances were calculated from all single ASVs classified within one family as per the best possible taxonomy using both RDP and SILVA.

SUPPLEMENTARY FIGURE 4 Relative and absolute abundances of commensal genera in porcine faecal microbiota. **(A)** Relative abundances of bacterial families obtained by standard analysis of 16S rRNA gene sequencing. **(B)** Same data as in panel A after GCN correction. **(C)** Same data as in panel B after integrating total cell counts obtained from flow cytometry and **(D)** after calculating absolute abundances of bacterial genera from spike-in DNA after sequencing. Significantly changed relative and absolute abundances of single genera are shown in boxplots. Mean values over all six animals are indicated. Cumulative abundances were calculated from all single ASVs classified within one genus as per the best possible taxonomy using both RDP and SILVA (# *p* < 0.05 after Paired Wilcoxon Signed Rank Sum Test, *n* = 6).
