# Supplementary figures and images for: Absolute abundance calculation enhances the significance of microbiome data in antibiotic treatment studies
Source: Front Microbiol. 2025 Mar 24;16:1481197. doi: 10.3389/fmicb.2025.1481197 (PMC11973300; doi:10.3389/fmicb.2025.1481197)

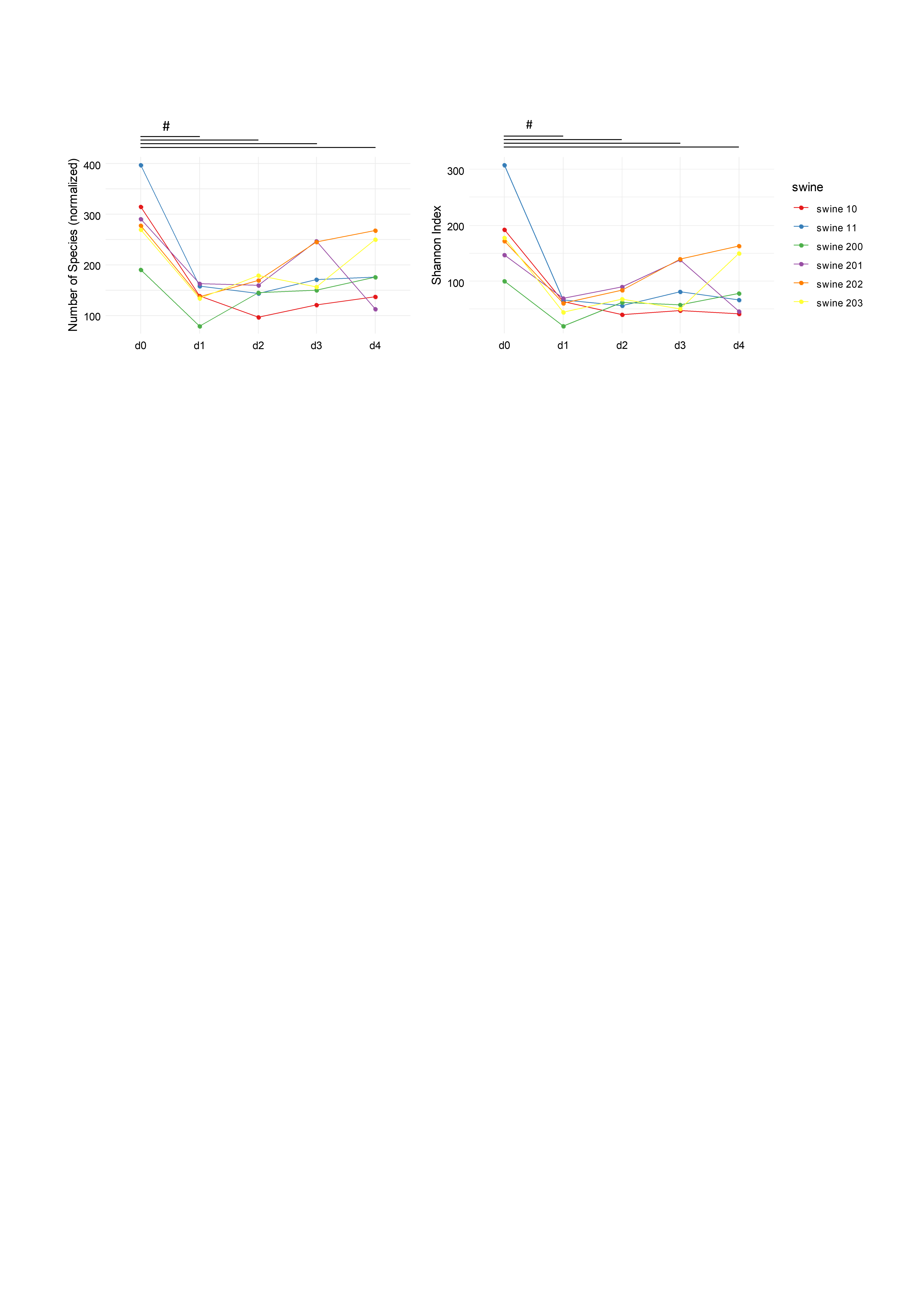

Supplement: SUPPLEMENTARY FIGURE 1 — α-diversity of faecal microbiota upon tylosin treatment. (A) Normalized number of species and (B) Shannon effective numbers of faecal microbiota obtained by standard analysis of 16S rRNA gene sequencing data is shown a line plot. Individual piglets are indicated by the corresponding colours. Cumulative abundances were calculated from all single ASVs classified within one family as per the best possible taxonomy using both RDP and SILVA (# p < 0.05 after Paired Wilcoxon Signed Rank Sum Test, n = 6). [file Image_1.TIF]

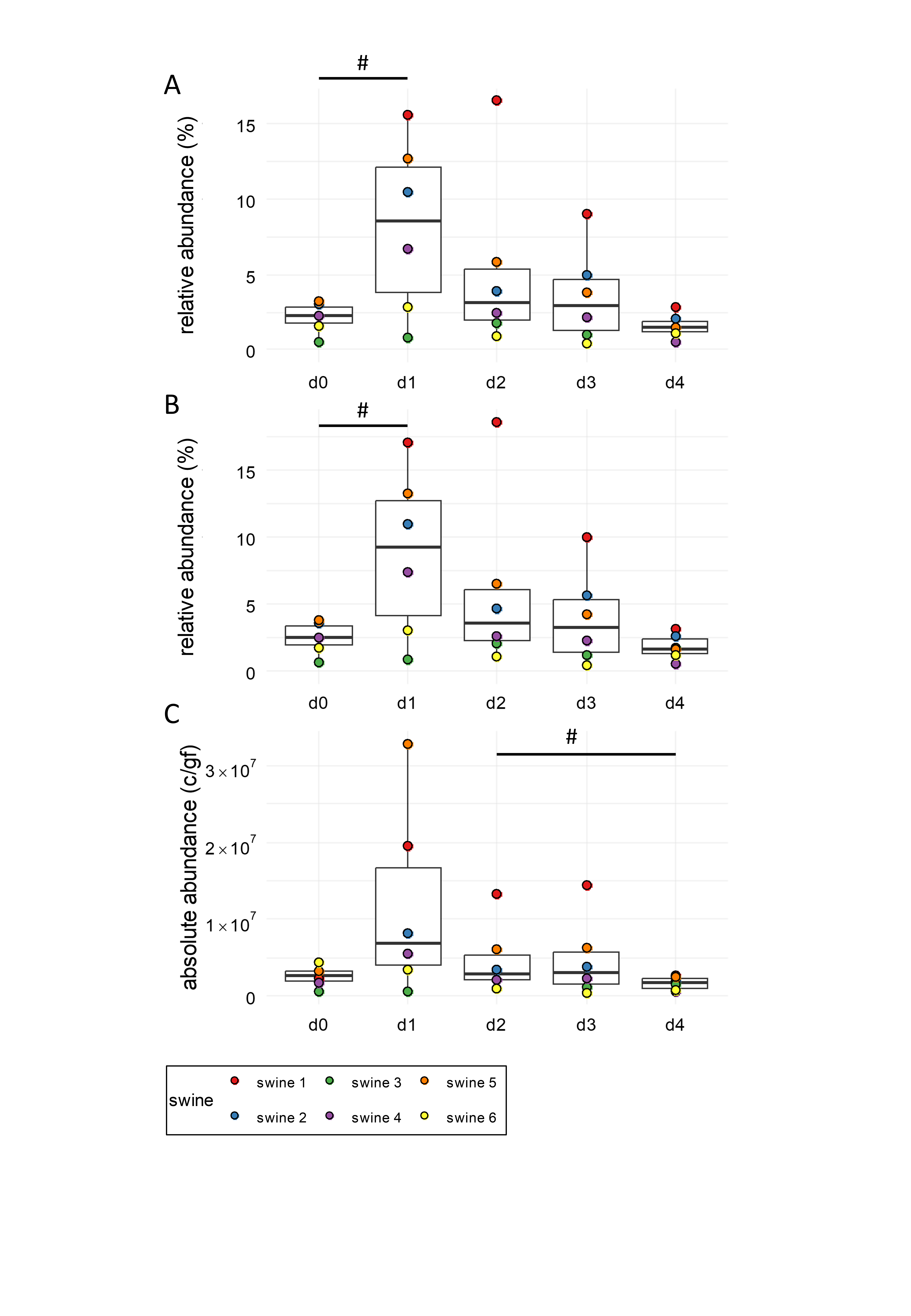

Supplement: SUPPLEMENTARY FIGURE 2 — Correction of 16S rRNA GCN and integration of bacterial cell counts into relative abundance analyses of the phylum Pseudomonadota. (A) Relative abundances of Pseudomonadota were obtained by standard analysis of 16S rRNA gene sequencing data. (B) Same data set as panel A, but corrected for GCN of the 16S rRNA genes. (C) Same data as in panel B, but absolute abundances of Pseudomonadota were obtained by integration of bacterial cells counts via flow cytometry. Relative and absolute abundances in the faecal microbiota of each animal are shown in boxplots. Cumulative abundances were calculated from all single ASVs classified within one phylum as per the best possible taxonomy using both RDP and SILVA (# p < 0.05 after Paired Wilcoxon Signed Rank Sum Test, n = 6). [file Image_2.TIF]

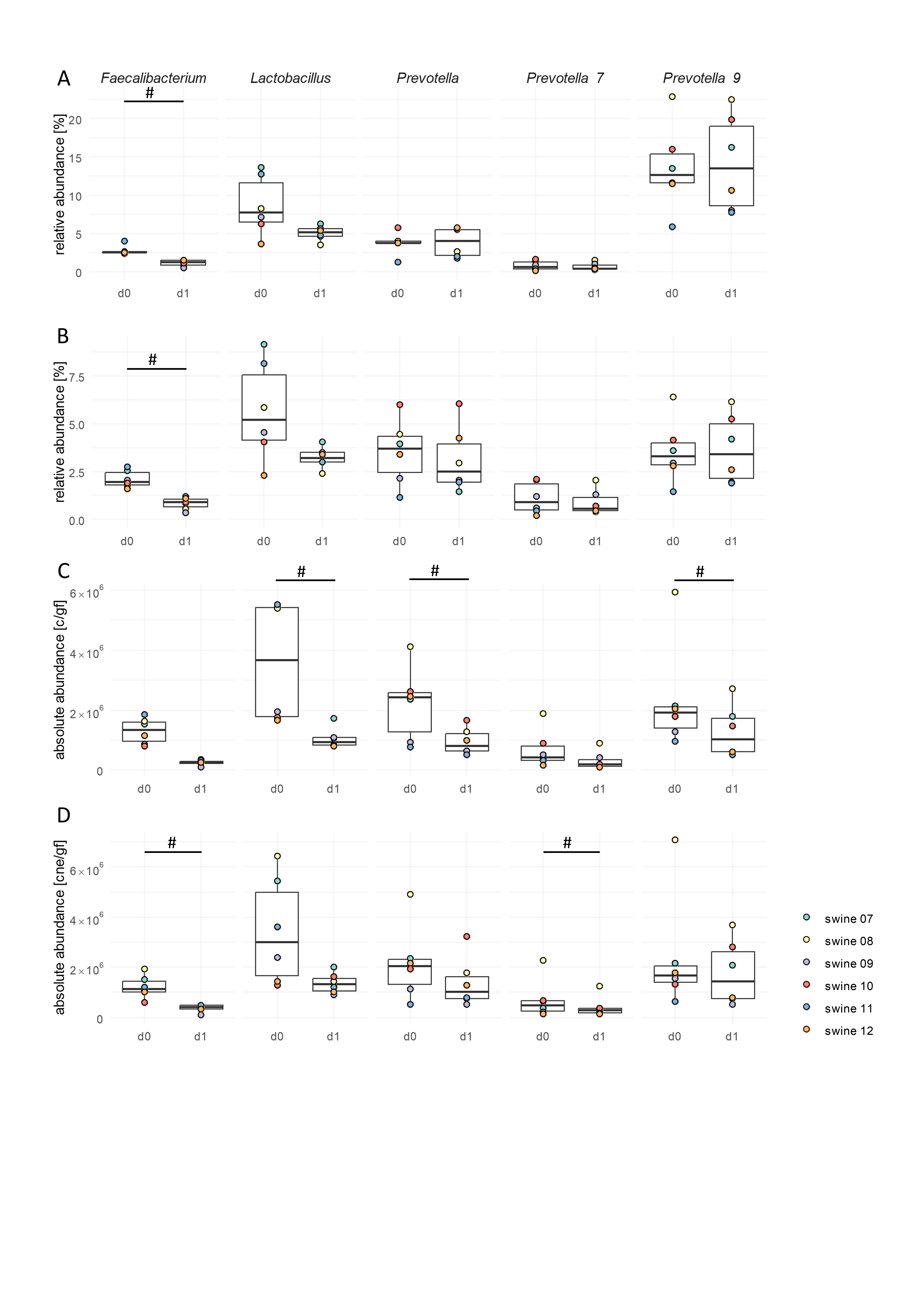

Supplement: SUPPLEMENTARY FIGURE 4 — Relative and absolute abundances of commensal genera in porcine faecal microbiota. (A) Relative abundances of bacterial families obtained by standard analysis of 16S rRNA gene sequencing. (B) Same data as in panel A after GCN correction. (C) Same data as in panel B after integrating total cell counts obtained from flow cytometry and (D) after calculating absolute abundances of bacterial genera from spike-in DNA after sequencing. Significantly changed relative and absolute abundances of single genera are shown in boxplots. Mean values over all six animals are indicated. Cumulative abundances were calculated from all single ASVs classified within one genus as per the best possible taxonomy using both RDP and SILVA (# p < 0.05 after Paired Wilcoxon Signed Rank Sum Test, n = 6). [file Image_4.TIF]
